# Bilateral adrenocortical carcinoma in a patient with multiple endocrine neoplasia type 1 (MEN1) and a novel mutation in the MEN1 gene

**DOI:** 10.1186/1477-7819-9-6

**Published:** 2011-01-25

**Authors:** John E Griniatsos, Nikoletta Dimitriou, Athanassios Zilos, Stratigoula Sakellariou, Konstantinos Evangelou, Smaragda Kamakari, Penelope Korkolopoulou, Gregory Kaltsas

**Affiliations:** 1 1st Department of Surgery, Medical School, University of Athens, Athens, Greece; 2 Division of Endocrinology, Department of Pathologic Physiology Medical School, University of Athens, Athens, Greece; 3 Department of Pathology, Medical School, University of Athens, Athens, Greece; 4 BioGenomica Genetic Tests Center, Athens, Greece

## Abstract

The incidence of adrenal involvement in MEN1 syndrome has been reported between 9 and 45%, while the incidence of adrenocortical carcinoma (ACC) in MEN1 patients has been reported between 2.6 and 6%. In the literature data only unilateral development of ACCs in MEN1 patients has been reported. We report a 31 years-old female MEN1-patient, in whom hyperplasia of the parathyroid glands, prolactinoma, non functioning pancreatic endocrine carcinoma and functioning bilateral adrenal carcinomas were diagnosed. Interestingly, a not previously described in the literature data, novel germline mutation (p.E45V) in exon 2 of MEN1 gene, was detected. The association of exon 2 mutation of the MEN1 gene with bilateral adrenal carcinomas in MEN1 syndrome, should be further investigated.

## Introduction

Multiple endocrine neoplasia type 1 (MEN1) is an autosomal dominant disorder with penetrance reaching 100% with age [1].

It is characterized by parathyroid glands hyperplasia, anterior pituitary gland tumours and pancreatic islets tumours [2]. However, other endocrine and non-endocrine lesions, such as carcinoids of the bronchi [3], gastrointestinal tract [4] and thymus [5], lipomas, angiofibromas and collagenomas [6,7] can also occur with low frequency, while combinations of more than twenty different endocrine and non-endocrine tumours and lesions have been reported [8-12].

A simple definition of MEN 1 can not cover all index cases and all families. As a practical definition, sporadic MEN1 is characterised by the occurrence of primary tumours involving two of the three main MEN1 related endocrine tissues within a single patient, while familial MEN1 is defined as at least one MEN1 case plus at least one first degree relative with one of those three tumors [13].

The incidence of adrenal lesions in MEN1 patients varies between 9 and 45% [14-20] and they usually develop in patients with mutations in exons 2 and 10 [18]. Other authors stated that they are mainly unilateral (55-79%) [18,20-22], others addressed them as mainly bilateral (60%) [15], but all agree that the majority of these lesions are hyperplastic and nonfunctioning, causing minimal morbidity. Functioning tumors like pheochromocytoma or tumors causing hypercortisolemia and hyperaldosteronism are rare manifestations of MEN1 [23]. The incidence of adreonocortical carcinoma (ACC) in MEN1 patients has been reported between 2.6 [20]and 6% [17,18,22]. Although involvement of the adrenal gland has been reported in approximately 40% of MEN1 patients and has been found to represent bilateral hyperplasia, adenoma and in a few cases carcinoma, bilateral adrenal carcinoma has not been previously reported.

Herewith, we report a 31 years-old female patient with sporadic form of MEN1 in whom functioning bilateral adrenocortical carcinomas were diagnosed. Interestingly enough, a novel germline mutation in exon 2 of MEN1 gene, was detected.

### Case Report

A 31 years-old female patient with the preoperative diagnosis of MEN1, was referred by the Endocrinologists to the 1^st ^Department of Surgery, for further evaluation and treatment.

On clinical examination, centripetal obesity, moon face, fat deposition over the thoracocervical spine (buffalo hump), excessive terminal hair in various parts of her body and four angiofibromas in her face, were observed. Her arterial blood pressure was moderately abnormal (155/80 mmHg), while she reported menstrual irregularity (infrequent uterine bleeding) and easy bruising. She was suffering from diabetes mellitus since 2002 and she was currently on glargine insulin, whereas she reported having been prescribed Dopamine agonists for her pituitary prolactinoma lesion. Her family history was insignificant

**Figure 1:**
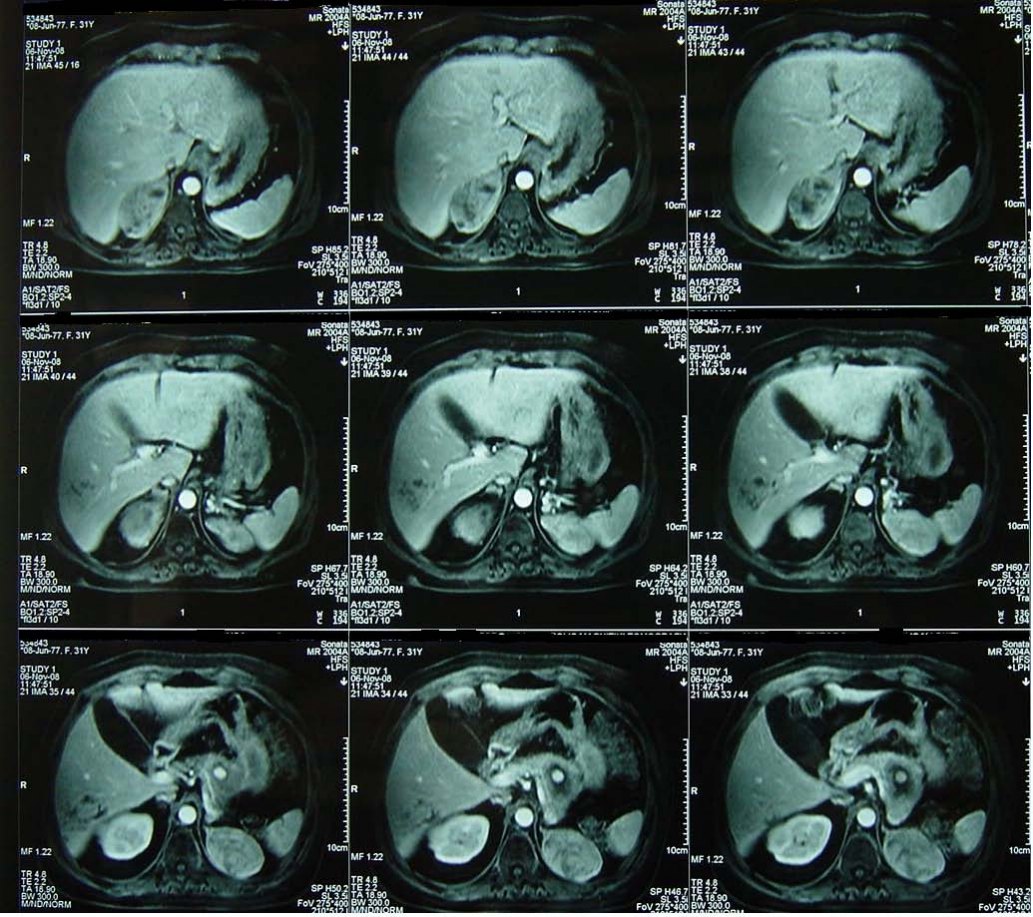
**MRI scan of the upper abdomen disclosing bilateral adrenal tumors**.

The preoperative imaging investigation of the patient included: magnetic resonance imaging (MRI) of the head disclosing a 1.4 × 1.3 cm pituitary adenoma, cervical ultrasound and Tc99 m Sestamibi-scans both disclosing hyperplasia of the parathyroid glands, abdominal MRI scan and endoscopic ultrasound (EUS) both disclosing a 4.5 × 3.0 cm tumor in the body and tail of the pancreas, fine needle aspiration biopsy (FNAB) of the pancreatic tumor under EUS guidance concluding in histological findings compatible to neuroendocrine tumor, somatostatin receptor imaging with Indium 111 (OctreoScan^®^) disclosing radionuclide material uptake in the left upper quadrant of the abdominal cavity at some point between the left lobe of the liver and the spleen, while in the abdominal MRI scan bilateral adrenal tumors (right adrenal 8.0 Χ 4.5 cm and left adrenal 7.5 × 5.5 cm) (Figure 1), as well as a fully calcified ectopic left kidney into the pelvis, were detected. Finally, a DMSA renal scan disclosed only 17% renal function of the ectopic left kidney.

The preoperative hormonal evaluation of the patient is presented in Table 1. She was further submitted to an Overnight Dexamethasone Suppression Test (ODST), which failed to suppress the morning cortisol levels- which were 627 nM/L (< 50 nM/L), whereas the Urinary Free Cortisol levels were elevated- 473 μg/24h (55-286), signifying hyper-cortisolism. The ODST, in concordance with the ACTH level, unmasked a Cushing syndrome caused due to adrenal production of cortisol. Moreover, the possibility of adrenocortical carcinoma was considered high in the light of the high levels of androgens. Due to moderate high levels of arterial blood pressure (155/80 mmHg), she had her 24 hours urine output collected. 800mL of urine were collected and the laboratory results were as follows: Adrenaline 3 μg/24h (1.7-22.4), Noradrenaline 29 μg/24h (12-85), Metanefrine 337 μg/24h (100-800), Normetanefrine 298 μg/24h (88-444), VMA 4 μg/24h (1.8-6.7) and Dopamine 47 μg/24h (65-400).

**Table 1:** Preoperative and postoperative serum hormonal evaluation of the patient

Parameter	Normal values	Preoperative value	Postoperative value
ACTH	1-50 pg/ml	0.23	
Cortisol	260-720 nM	834	
Prolactin	2.7-20 ng/ml	25.7	
Calcium	8.6-10.2 mg/dl	9.8	
PTH	8-76 pg/ml	107	
GH	0-10 μIU/ml	12	
IGF	114-492 ng/ml	847	
FSH	1.8-9.4 mIU/ml	0.42	
LH	0.8-10.4 mIU/ml	1.1	
TSH	0.3-4 mU/l	1.65	
Testosterone	0.1-0.8 ng/ml	2	0.1
Δ4 Androstendione	0.5-4.7 ng/ml	14.7	0.38
DHEAs	99-340 μg/dl	950	0.01
Phosphorus	2.7-4.5 mg/dl	2.7	
CgA	19-98 ng/ml	187	
CA-125	< 30 U/ml	227	40

After the completion of the imaging investigation and the laboratory evaluation, the diagnosis of sporadic form of MEN1 syndrome consisting of: (i) a 1.4 × 1.3 cm prolactine producing pituitary adenoma, (ii) primary hyperparathyroidism due to hyperplasia of the parathyroid glands, (iii) a 4.5 × 3.0 cm non functioning endocrine tumor in the body and tail of the pancreas and (iv) functioning bilateral adrenal tumors, was established.

The patient was submitted to an exploratory laparotomy through a bilateral subcostal incision. She underwent distal pancreatectomy, splenectomy, bilateral adrenalectomy and nephrectomy of the ectopic left kidney. Parathyroidectomy was postponed until the development of clinical or laboratory findings compatible to hyperparathyroidism. The patient had an uneventful postoperative recovery and she was discharge on the 7^th ^postoperative day.

The histological findings were as follows: Distal pancreas (size 9.5 × 5.1 × 2.8 cm). Grossly, the pancreatic tissue exhibited multiple cystic and hemorrhagic lesions ranging from 0.3 to 2 cm in maximum diameter. In the peripancreatic fat five lymphnodes measuring 0.4 to 0.6 cm, were detected. Microscopically, the pancreatic lesions corresponded to neoplastic tissue consisting of uniform neoplastic cells with scant eosinophilic cytoplasm and stippled nuclei, arranged in nests and trabecula. There was mild to moderate nuclear atypia and the mitotic figures were less than 2 per 10 HPF (magnification X40). Neoplastic emboli in pancreatic vessels, as well as tumor nests in the peripancreatic fat were also evident. The tumor appeared to extend within less than 0.3 cm of the surgical margins. The resected lymphnodes were free of metastatic deposits. The neoplastic cells exhibited immunopositivity for neuroendocrine markers (chromogranin and synaptophysin), while the proliferation index assessed by Ki-67 immunohistochemistry was about 5%. Taking into account the above histological observations (especially that the proliferation index was about 5%) as well as the data from the literature, the diagnosis of well-differentiated endocrine carcinoma of the pancreas was established.

**Figure 2:**
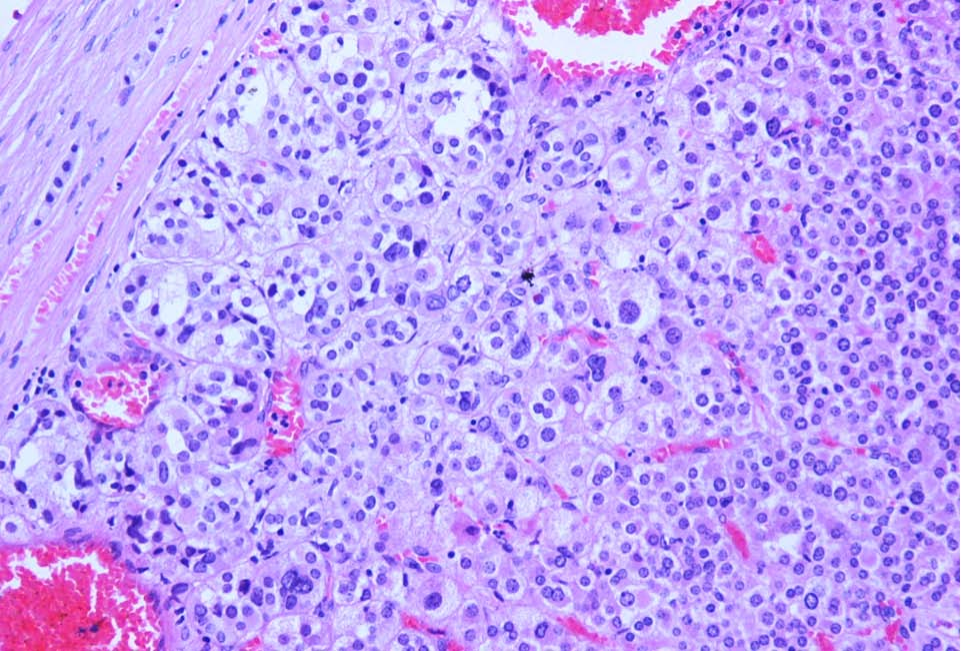
**Histological section (H&E counterstain) of the adrenocortical neoplasm in the left adrenal gland, depicting cells with moderate to marked nuclear pleomorphism (magnification X200)**.

Both adrenals glands. The left one weighed 250 gr, measured 10.1 × 7.3 × 4.5 cm and upon sectioning, a well circumscribed brownish tumor with central calcification sizing 7.5 × 5.5 × 4.1 cm, was found. The right adrenal gland weighed 120gr and measured 10.5 × 5.5 × 3.7 cm. Gross examination revealed the presence of a tumor measuring 8 × 4 × 3 cm, with features similar to those of the left gland, but without central calcification. Histologically, both adrenal tumors showed features of adrenocortical neoplasms. The neoplastic population consisted of round to oval cells, with scant eosinophilic cytoplasm and moderate to marked nuclear pleomorphism, arranged in a loose growth pattern (Figure 2). Abnormal caryokinesis and invasion of venous and sinusoid vessels were also observed. Necrotic areas were present only in the tumor of the left adrenal gland. Immunohistochemically, the cells were MelanA and synaptophysin immunopositive. No immunostaining for cytoceratin, chromogranin, EMA and CEA was observed. The remaining non-neoplastic adrenal tissue showed nodular hyperplasia. Based on the above findings, both tumors corresponded to adrenocortical neoplasms with malignant potential, according to Weiss's criteria.

Following these results, the patient as well as her mother and her sister were submitted to genetic test for MEN1. A germline p.E45V mutation in exon 2 of the MEN1 gene was detected, while none of the rest members of the family expressed any genetic abnormality.

Due to the bilateral adrenalectomy she was prescribed on Hydrocortisone and Flurohydrocortisone repletion therapy. Her postoperative hormonal evaluation, soon after having been discharged, is presented in Table 1.

## Discussion

In the present report we describe a young female MEN1-patient, in whom apart from the prolactine producing pituitary adenoma, the hyperplasia of the parathyroid glands and the well-differentiated non functioning pancreatic endocrine carcinoma, functioning bilateral adrenocortical carcinomas were diagnosed. A not previously described, novel germline mutation (p.E45V) in exon 2 of MEN1 gene was detected in the postoperative genetic test for MEN1.

MEN1 is a tumor suppressor gene located at chromosome 11q13 loci, encoding a 67 kD nuclear protein of 610 amino acids, called menin [24]. Various types of mutations scattered throughout the 10 coding exons of the MEN1 gene inactivate the MEN1 gene.

Knudson's two-hit model for tumorigenesis [10] addresses that the first hit can take place either in germline or in somatic tissue and compromise a small number of bases. The mutations are distributed across the MEN1 reading frame, resulting in a great deal of novel mutations, approximately 50%, in the index cases. Mostly, they result in premature truncation of DNA sequence, inactivating menin protein. The second hit is usually a large chromosomal deletion, which lives no normal functional allele. The second hit is in somatic tissue and in most cases occurs postnataly.

The first clinical manifestation of MEN1 is hyperparathyroidism, mainly due to multiglandular hyperplasia, which usually affects more than 95% of all MEN1 patients [25]. Although there is no doubt that total parathyroidectomy constitutes the treatment of choice for symptomatic hypercalcaemic MEN1 patients [2], the decision for the timing for the parathyroid surgery should take under account the severity of the PHPT symptoms, the circulating PTH and calcium levels, the presence of MEN1-associated endocrinopathies, especially the Zollinger-Ellison syndrome (ZES) and the patient's age [26]. Since the patient we describe, was asymptomatic, had no evidence of ZES, meeting only one (the age) of the proposed by the NIH criteria for the treatment of asymptomatic primary hyperparathyroidism [27], the parathyroidectomy was postponed for the future.

Pancreatic tumours occur in about 30-75% of MEN1 patients and are the second commonest clinical manifestation of MEN1 [28]. One third of pancreatic tumours are non functioning and clinically silent, but the majority of them produce excessive amounts of hormones such as gastrin, insulin, glucagon, somatostatin, neurotensin or vasoactive intestinal polypeptide and are associated with distinct clinical syndromes [2]. Pancreatic tumours can be multiple and scattered throughout the whole pancreas, ranging in size from micro-adenomas (slightly larger than unaffected islets) to macro-adenomas larger than 0.5 cm [26]. Endoscopic ultrasound (EUS) constitutes the most sensitive imaging method for the detection of small pancreatic endocrine tumours in asymptomatic MEN1 patients (sensitivity > 75%), while the combination of EUS with Octreoscan scintigraphy, increases the pancreatic tumoural detection rate to 90% [29]. As general recommendation, patients with positive imaging studies and no evidence of unresectable metastases, should undergo surgical exploration with intraoperative ultrasound [10]. Surgery is always indicated for hormonal producing tumours, while for asymptomatic patients pancreatic surgery can be decided when the size of the lesion approaches 2 cm [26]. It is generally accepted that large tumours in the pancreatic body or tail should be treated by distal pancreatectomy and splenectomy [30]. The preoperative laboratory and imaging with EUS and Octreoscan work up of the patient we describe, disclosed a non hormonal producing but greater than 2 cm pancreatic tail tumour with no evidence of metastatic disease. Thus, the patient underwent an exploratory laparotomy, the intraoperative ultrasonography did not disclosed any other pancreatic lesion and distal pancreatectomy and splenectomy were performed.

The incidence of pituitary adenomas in MEN1 patients varies between 10 and 60% [13], while their symptoms depend on the type and the amount of the pituitary hormone secretion and/or the compression effects due to the size of the tumour [26]. Pituitary tumours can be successfully managed by drug therapy, reserving surgery and/or radiotherapy for large tumours or irresectable residual disease [2]. The lack of compression symptoms explains why the patient we describe, was treated conservatively.

Regarding the adrenocortical lesions, DNA analysis for allelic loss at the MEN1 locus shown no loss of heterozygosity [15,31], probably suggesting that they are not a direct result of inactivation of the MEN1 gene.

Since the malignant potential of MEN1-related adrenal neoplasia is of important clinical significance, close biochemical and radiologic follow-up is recommended. Newly diagnosed adrenal lesions should undergone a control investigation after 6 months and in case of a stable lesion, control intervals of 2 years seem to be sufficient [32]. Adrenal lesions smaller than 3 cm are usually asymptomatic and endocrine-inactive with low malignant potential [32]. Although general agreement does not exist, several authors [2,18,22] recommend that adrenocortical tumours greater than 3 cm in diameter or growing lesion [32] should be surgically removed because of their malignant potential. Because both adrenal lesions in the presented case were greater than 3 cm, bilateral adrenalectomy was performed.

In the patient we describe, a non functional, fully calcified ectopic pelvic kidney was also detected and surgically removed. Since ectopic kidneys are more susceptible to diseases such as nephrolithiasis, hydronephrosis, injury of aberrant vessels or overlying abdominal viscera and nerves or malignancy than the normally positioned ones, a non functional pelvic kidney requires removal [33].

Preoperatively, the patient we describe, had elevated (7.5-fold) serum CA-125 levels. It is well known the high false positive rate and the poor sensitivity and specificity of the CA-125. Although cardiovascular, lung and chronic liver diseases are the most frequent diagnoses in patients with increased CA-125, other intra-abdominal non-malignant non-hepatic diseases can also cause elevation of the CA-125 [34], as in the present case.

Many proteins that localize to the nucleus contain a polybasic motif, the Nuclear Localization Signal (NLS), which is necessary for proper nuclear targeting. Menin contains two NLSs. Both NLS-1 (amino acid 479-497) and NLS-2 (amino acids 588-608) are present in the C-fourth of menin [35]. Menin binds to JunD transcription factor, a member of Activator Protein 1 family. JunD regulates transcription from certain promoters by binding to TRE consensus. Menin-JunD interaction involves the N-terminus (amino acids 1-40) and midregion (amino acids 323-428) of Menin and requires the N-terminus of JunD (amino acids 8-70). Menin's tumor suppressor involves direct binding to JunD and inhibition of JunD activated transcription [36].

The patient we describe harbors the germline p.E45V mutation of the MEN1 gene which, to the best of our knowledge, has not been previously described in the literature data. It is a point mutation in exon 2 of MEN1 gene, in nucleotide 134 substituting Adenosine to Thymine, changing the Glutamic acid (GAG) to Valine (GTC) in the menin molecule. Although, pathogenetic mutations of glutamic acid either to lysine (p.E45K) or glykine (p.E45G) or alanine (p.E45A), have been previously described [37], the close proximity of this amino acid (amino acid 45) to the area (amino acids 1-40) of menin molecule responsive for Menin-JunD interaction, poses a possible explanation of the present tumorgenesis by disrupting Menin-JunD interaction, finally affecting the tumor suppression function.

In conclusion, we present a MEN1 patient with the novel p.E45V mutation in the exon 2 of the MEN1 gene, in whom bilateral ACC was detected. Whether this novel mutation predisposed to bilateral ACC development or whether bilateral ACC can also occur in MEN1 patients, remains to be proved.

## Competing interests

The authors declare that they have no competing interests.

## Authors' contributions

JG performed the operation and contributed to the preparation of the manuscript; ND assisted to the operation and wrote the manuscript; AZ prepared preoperatively and followed-up postoperatively the patient, wrote the manuscript and reviewed the literature; SS and KE performed the histopathology; SK performed the genetic test; PK performed the histopathology and immunohistochemistry; GK diagnosed the case, checked the final version of the manuscript and made significant comments. All authors read and approved the final manuscript.
